# *r*-2,*c*-6-Di­phenyl­piperidine

**DOI:** 10.1107/S1600536813020382

**Published:** 2013-08-03

**Authors:** V. Maheshwaran, S. Abdul Basheer, A. Akila, S. Ponnuswamy, M. N. Ponnuswamy

**Affiliations:** a Centre of Advanced Study in Crystallography and Biophysics, University of Madras, Guindy Campus, Chennai 600 025, India; b Department of Chemistry, Government Arts College (Autonomous), Coimbatore 641 018, India

## Abstract

In the title compound, C_17_H_19_N, the piperidine ring adopts a chair conformation. The phenyl rings substituted at the 2- and 6-positions of the piperidine ring subtend dihedral angles of 81.04 (7) and 81.10 (7)° with the best plane of the piperidine ring. The crystal packing features C—H⋯π inter­actions.

## Related literature

For the biological activity of piperidine derivatives, see: Aridoss *et al.* (2009 ▶); Boehringer & Söhne GmbH (1961 ▶); Jain *et al.* (2005 ▶); Kubota *et al.* (1998 ▶); Mobio *et al.* (1989 ▶); Rubiralta *et al.* (1991 ▶). For the synthesis of the title compound, see: Ponnuswamy *et al.* (2002 ▶). For puckering parameters, see: Cremer & Pople (1975 ▶). For asymmetry parameters, see: Nardelli (1983 ▶).
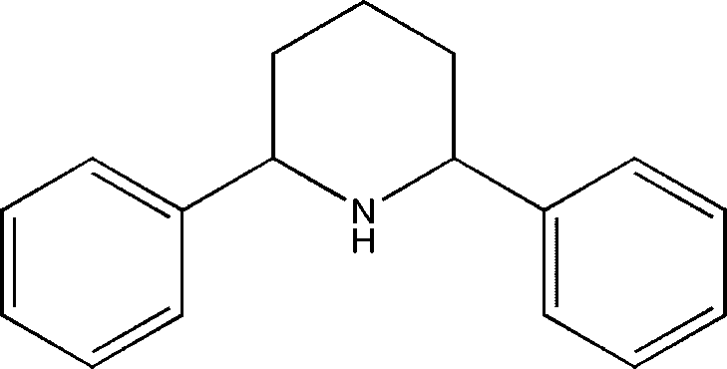


## Experimental

### 

#### Crystal data

C_17_H_19_N
*M* *_r_* = 237.33
Triclinic, 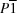

*a* = 5.6450 (9) Å
*b* = 11.2255 (17) Å
*c* = 11.5281 (17) Å
α = 73.911 (9)°
β = 89.898 (9)°
γ = 81.466 (9)°
*V* = 693.53 (18) Å^3^
*Z* = 2
Mo *K*α radiation
μ = 0.07 mm^−1^
*T* = 293 K
0.21 × 0.19 × 0.18 mm

#### Data collection

Bruker SMART APEXII CCD diffractometer
Absorption correction: multi-scan (*SADABS*; Bruker, 2008 ▶) *T*_min_ = 0.986, *T*_max_ = 0.988
9781 measured reflections
2813 independent reflections
2113 reflections with *I* > 2σ(*I*)
*R*_int_ = 0.032

#### Refinement

*R*[*F* ^2^ > 2σ(*F* ^2^)] = 0.043
*wR*(*F* ^2^) = 0.125
*S* = 1.06
2813 reflections
167 parameters
H atoms treated by a mixture of independent and constrained refinement
Δρ_max_ = 0.11 e Å^−3^
Δρ_min_ = −0.20 e Å^−3^

### 

Data collection: *APEX2* (Bruker, 2008 ▶); cell refinement: *SAINT* (Bruker, 2008 ▶); data reduction: *SAINT*; program(s) used to solve structure: *SHELXS97* (Sheldrick, 2008 ▶); program(s) used to refine structure: *SHELXL97* (Sheldrick, 2008 ▶); molecular graphics: *ORTEP-3 for Windows* (Farrugia, 2012 ▶); software used to prepare material for publication: *SHELXL97* and *PLATON* (Spek, 2009 ▶).

## Supplementary Material

Crystal structure: contains datablock(s) global, I. DOI: 10.1107/S1600536813020382/bt6919sup1.cif

Structure factors: contains datablock(s) I. DOI: 10.1107/S1600536813020382/bt6919Isup2.hkl

Supplementary material file. DOI: 10.1107/S1600536813020382/bt6919Isup3.cml

Additional supplementary materials:  crystallographic information; 3D view; checkCIF report

## Figures and Tables

**Table 1:** Hydrogen-bond geometry (Å, °) *Cg*1 and *Cg*2 are the centroids of the C13–C18 and C7–C12 rings, respectively.

*D*—H⋯*A*	*D*—H	H⋯*A*	*D*⋯*A*	*D*—H⋯*A*
C3—H3*A*⋯*Cg*1^i^	0.97	3.00	3.719 (2)	132
C10—H10⋯*Cg*1^ii^	0.93	3.01	3.760 (2)	139
C16—H16⋯*Cg*2^iii^	0.93	3.03	3.799 (2)	141

Symmetry codes: (i) 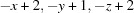; (ii) 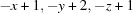; (iii) 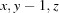.

**Fig. 1.**
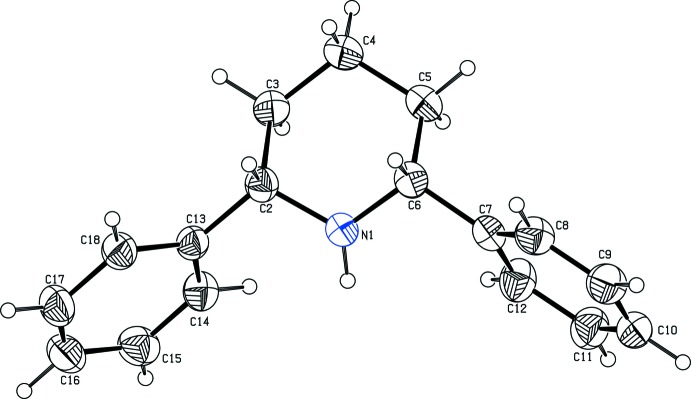
The molecular structure of the title compound, showing the atomic numbering and displacement ellipsoids drawn at 30% probability level.

**Fig. 2.**
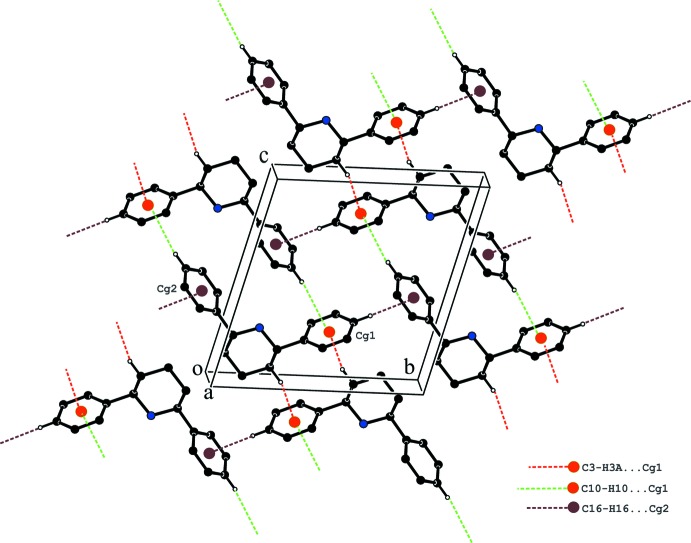
The crystal packing of the molecules viewed down a-axis.

## supplementary crystallographic information

### 1. Comment

Piperidines are valuable heterocyclic compounds found in natural substances and pharmaceutical products (Rubiralta *et al.*, 1991; Jain *et al.*, 2005; Kubota *et al.*, 1998). Several 2,6-substituted piperidine derivatives were found to be useful as tranquilisers (Boehringer & Söhne GmbH, 1961) and possess a wide range of biological activities such as antiviral, antimalarial, antibacterial and antifungal activities (Aridoss *et al.*, 2009, Mobio *et al.*, 1989). In view of the above importance, the crystallographic study of the title compound has been carried out to establish its molecular structure.

The *ORTEP* plot of the molecule is shown in Fig. 1. The piperidine ring adopts a chair conformation with puckering parameters (Cremer & Pople, 1975) and asymmetry parameters (Nardelli, 1983): q_2_=0.0420 (15) Å, q_3_ = -0.5799 (15) Å, φ_2_ = 190 (2)° and Δ_s_ (N1& C4)= 0.75 (12)°. The phenyl rings at 2,6-positions of the piperidine ring occupy equatorial positions. The corresponding torsion angles are [C13—C2—C3—C4] -178.62 (11)° & [C4—C5—C6—C7] 177.89 (12)°, respectively. The dihedral angle between the two phenyl rings is 60.0 (7)°. The phenyl rings [C7—C12 & C13—C18] are twisted away from the best plane of the piperidine moiety by 81.04 (7)° & 81.10 (7)°, respectively. The molecules in the unit cell are connected by C—H ···π interactions (Fig. 2 & Table. 1; Cg1 is the centroid of the ring C13 to C18 and Cg2 is the centroid of the ring C7 to C12).

### 2. Experimental

A mixture of piperidin-4-one (10 m*M*) and 80% hydrazine hydrate (3.1 ml) in diethylene glycol (100 ml) was heated on a steam bath for 2 hrs (Ponnuswamy *et al.*, 2002). Potassium hydroxide pellets (2.8 g) were added to the mixture and the contents were allowed to reflux vigorously on a heating mantle for another 2 hrs and the reaction mixture was cooled. The product formed was filtered and recrystallized from ethanol.

### 3. Refinement

All H atoms were found in a difference map. Nevertheless, those bonded to C were positioned geometrically (C–H = 0.93–0.98 Å) and allowed to ride on their parent atoms, with *U*_iso_(H) = 1.2*U*_eq_(C). The H atom bonded to N was freely refined.

### Crystal data

**Table:**

C_17_H_19_N	*Z* = 2
*M**_r_* = 237.33	*F*(000) = 256
Triclinic, *P*1	*D*_x_ = 1.136 Mg m^−^^3^
Hall symbol: -P 1	Mo *K*α radiation, λ = 0.71073 Å
*a* = 5.6450 (9) Å	Cell parameters from 2113 reflections
*b* = 11.2255 (17) Å	θ = 1.8–26.6°
*c* = 11.5281 (17) Å	µ = 0.07 mm^−^^1^
α = 73.911 (9)°	*T* = 293 K
β = 89.898 (9)°	Block, white
γ = 81.466 (9)°	0.21 × 0.19 × 0.18 mm
*V* = 693.53 (18) Å^3^	

### Data collection

**Table:**

Bruker SMART APEXII CCD diffractometer	2813 independent reflections
Radiation source: fine-focus sealed tube	2113 reflections with *I* > 2σ(*I*)
Graphite monochromator	*R*_int_ = 0.032
ω and φ scans	θ_max_ = 26.6°, θ_min_ = 1.8°
Absorption correction: multi-scan (*SADABS*; Bruker, 2008)	*h* = −7→7
*T*_min_ = 0.986, *T*_max_ = 0.988	*k* = −13→14
9781 measured reflections	*l* = −14→14

### Refinement

**Table:**

Refinement on *F*^2^	Primary atom site location: structure-invariant direct methods
Least-squares matrix: full	Secondary atom site location: difference Fourier map
*R*[*F*^2^ > 2σ(*F*^2^)] = 0.043	Hydrogen site location: inferred from neighbouring sites
*wR*(*F*^2^) = 0.125	H atoms treated by a mixture of independent and constrained refinement
*S* = 1.06	*w* = 1/[σ^2^(*F*_o_^2^) + (0.0582*P*)^2^ + 0.0755*P*] where *P* = (*F*_o_^2^ + 2*F*_c_^2^)/3
2813 reflections	(Δ/σ)_max_ < 0.001
167 parameters	Δρ_max_ = 0.11 e Å^−^^3^
0 restraints	Δρ_min_ = −0.20 e Å^−^^3^

### Special details

**Table:**

Geometry. All e.s.d.'s (except the e.s.d. in the dihedral angle between two l.s. planes) are estimated using the full covariance matrix. The cell e.s.d.'s are taken into account individually in the estimation of e.s.d.'s in distances, angles and torsion angles; correlations between e.s.d.'s in cell parameters are only used when they are defined by crystal symmetry. An approximate (isotropic) treatment of cell e.s.d.'s is used for estimating e.s.d.'s involving l.s. planes.
Refinement. Refinement of *F*^2^ against ALL reflections. The weighted *R*-factor *wR* and goodness of fit *S* are based on *F*^2^, conventional *R*-factors *R* are based on *F*, with *F* set to zero for negative *F*^2^. The threshold expression of *F*^2^ > σ(*F*^2^) is used only for calculating *R*-factors(gt) *etc*. and is not relevant to the choice of reflections for refinement. *R*-factors based on *F*^2^ are statistically about twice as large as those based on *F*, and *R*- factors based on ALL data will be even larger.

### Fractional atomic coordinates and isotropic or equivalent isotropic displacement parameters (Å^2^)

**Table:**

	*x*	*y*	*z*	*U*_iso_*/*U*_eq_	
C2	0.3110 (2)	0.28559 (11)	0.14071 (11)	0.0526 (3)	
H2	0.4718	0.2820	0.1084	0.063*	
C3	0.1318 (3)	0.28661 (13)	0.04187 (13)	0.0649 (4)	
H3A	−0.0288	0.2927	0.0720	0.078*	
H3B	0.1375	0.3595	−0.0264	0.078*	
C4	0.1868 (3)	0.16823 (14)	0.00071 (13)	0.0762 (4)	
H4A	0.3376	0.1680	−0.0398	0.091*	
H4B	0.0619	0.1672	−0.0565	0.091*	
C5	0.2026 (3)	0.05172 (13)	0.10787 (13)	0.0680 (4)	
H5A	0.2539	−0.0222	0.0809	0.082*	
H5B	0.0452	0.0454	0.1407	0.082*	
C6	0.3782 (2)	0.05592 (11)	0.20619 (12)	0.0547 (3)	
H6	0.5379	0.0580	0.1730	0.066*	
C7	0.3915 (2)	−0.05597 (11)	0.31576 (12)	0.0525 (3)	
C8	0.5902 (3)	−0.14804 (13)	0.34202 (13)	0.0643 (4)	
H8	0.7197	−0.1406	0.2919	0.077*	
C9	0.5997 (3)	−0.25123 (14)	0.44165 (15)	0.0737 (4)	
H9	0.7353	−0.3123	0.4580	0.088*	
C10	0.4108 (3)	−0.26438 (14)	0.51663 (13)	0.0699 (4)	
H10	0.4176	−0.3341	0.5835	0.084*	
C11	0.2118 (3)	−0.17362 (14)	0.49196 (15)	0.0747 (4)	
H11	0.0829	−0.1816	0.5425	0.090*	
C12	0.2023 (3)	−0.07085 (13)	0.39271 (14)	0.0685 (4)	
H12	0.0662	−0.0101	0.3769	0.082*	
C13	0.2607 (2)	0.39961 (11)	0.18678 (11)	0.0501 (3)	
C14	0.0632 (3)	0.41659 (13)	0.25525 (13)	0.0618 (4)	
H14	−0.0411	0.3577	0.2714	0.074*	
C15	0.0190 (3)	0.51912 (14)	0.29970 (15)	0.0735 (4)	
H15	−0.1140	0.5289	0.3459	0.088*	
C16	0.1715 (3)	0.60761 (14)	0.27597 (15)	0.0754 (4)	
H16	0.1422	0.6768	0.3063	0.090*	
C17	0.3659 (3)	0.59283 (14)	0.20757 (15)	0.0734 (4)	
H17	0.4680	0.6527	0.1908	0.088*	
C18	0.4119 (3)	0.48965 (12)	0.16320 (12)	0.0604 (4)	
H18	0.5453	0.4804	0.1172	0.072*	
N1	0.30501 (19)	0.17179 (9)	0.24060 (10)	0.0527 (3)	
H1	0.397 (3)	0.1732 (13)	0.3027 (14)	0.066 (4)*	

### Atomic displacement parameters (Å^2^)

**Table:**

	*U*^11^	*U*^22^	*U*^33^	*U*^12^	*U*^13^	*U*^23^
C2	0.0521 (7)	0.0509 (7)	0.0556 (7)	−0.0122 (5)	0.0034 (6)	−0.0143 (6)
C3	0.0785 (9)	0.0594 (8)	0.0559 (8)	−0.0142 (7)	−0.0073 (7)	−0.0128 (6)
C4	0.1052 (12)	0.0700 (9)	0.0583 (8)	−0.0204 (8)	−0.0091 (8)	−0.0225 (7)
C5	0.0859 (10)	0.0597 (8)	0.0655 (9)	−0.0183 (7)	−0.0016 (7)	−0.0255 (7)
C6	0.0543 (7)	0.0504 (7)	0.0614 (8)	−0.0093 (5)	0.0064 (6)	−0.0183 (6)
C7	0.0547 (7)	0.0476 (7)	0.0595 (7)	−0.0083 (5)	0.0012 (6)	−0.0217 (6)
C8	0.0604 (8)	0.0652 (9)	0.0672 (9)	−0.0004 (7)	0.0016 (7)	−0.0231 (7)
C9	0.0791 (10)	0.0627 (9)	0.0734 (10)	0.0087 (7)	−0.0137 (8)	−0.0192 (8)
C10	0.0935 (11)	0.0570 (8)	0.0580 (8)	−0.0140 (8)	−0.0090 (8)	−0.0130 (7)
C11	0.0819 (11)	0.0673 (9)	0.0728 (10)	−0.0147 (8)	0.0162 (8)	−0.0145 (8)
C12	0.0646 (9)	0.0564 (8)	0.0788 (10)	−0.0017 (6)	0.0114 (7)	−0.0134 (7)
C13	0.0523 (7)	0.0469 (7)	0.0489 (7)	−0.0089 (5)	−0.0042 (5)	−0.0093 (5)
C14	0.0610 (8)	0.0571 (8)	0.0674 (8)	−0.0119 (6)	0.0053 (7)	−0.0160 (7)
C15	0.0747 (10)	0.0723 (10)	0.0744 (10)	−0.0002 (8)	0.0043 (8)	−0.0277 (8)
C16	0.0932 (12)	0.0606 (9)	0.0760 (10)	−0.0017 (8)	−0.0151 (9)	−0.0300 (8)
C17	0.0845 (11)	0.0602 (9)	0.0810 (10)	−0.0244 (8)	−0.0089 (9)	−0.0215 (8)
C18	0.0621 (8)	0.0595 (8)	0.0618 (8)	−0.0179 (6)	−0.0014 (6)	−0.0162 (6)
N1	0.0583 (6)	0.0468 (6)	0.0540 (6)	−0.0091 (5)	−0.0049 (5)	−0.0154 (5)

### Geometric parameters (Å, º)

**Table:**

C2—N1	1.4685 (16)	C9—C10	1.370 (2)
C2—C13	1.5064 (17)	C9—H9	0.9300
C2—C3	1.5217 (19)	C10—C11	1.372 (2)
C2—H2	0.9800	C10—H10	0.9300
C3—C4	1.5211 (19)	C11—C12	1.376 (2)
C3—H3A	0.9700	C11—H11	0.9300
C3—H3B	0.9700	C12—H12	0.9300
C4—C5	1.521 (2)	C13—C14	1.3857 (18)
C4—H4A	0.9700	C13—C18	1.3881 (17)
C4—H4B	0.9700	C14—C15	1.375 (2)
C5—C6	1.522 (2)	C14—H14	0.9300
C5—H5A	0.9700	C15—C16	1.380 (2)
C5—H5B	0.9700	C15—H15	0.9300
C6—N1	1.4648 (16)	C16—C17	1.367 (2)
C6—C7	1.5072 (18)	C16—H16	0.9300
C6—H6	0.9800	C17—C18	1.382 (2)
C7—C8	1.3789 (19)	C17—H17	0.9300
C7—C12	1.3867 (19)	C18—H18	0.9300
C8—C9	1.381 (2)	N1—H1	0.891 (16)
C8—H8	0.9300		
			
N1—C2—C13	109.73 (10)	C9—C8—H8	119.5
N1—C2—C3	108.14 (10)	C10—C9—C8	120.55 (14)
C13—C2—C3	113.09 (11)	C10—C9—H9	119.7
N1—C2—H2	108.6	C8—C9—H9	119.7
C13—C2—H2	108.6	C9—C10—C11	119.28 (14)
C3—C2—H2	108.6	C9—C10—H10	120.4
C4—C3—C2	111.00 (12)	C11—C10—H10	120.4
C4—C3—H3A	109.4	C10—C11—C12	120.17 (15)
C2—C3—H3A	109.4	C10—C11—H11	119.9
C4—C3—H3B	109.4	C12—C11—H11	119.9
C2—C3—H3B	109.4	C11—C12—C7	121.37 (14)
H3A—C3—H3B	108.0	C11—C12—H12	119.3
C3—C4—C5	110.73 (12)	C7—C12—H12	119.3
C3—C4—H4A	109.5	C14—C13—C18	118.18 (12)
C5—C4—H4A	109.5	C14—C13—C2	120.72 (11)
C3—C4—H4B	109.5	C18—C13—C2	121.09 (12)
C5—C4—H4B	109.5	C15—C14—C13	121.03 (13)
H4A—C4—H4B	108.1	C15—C14—H14	119.5
C4—C5—C6	111.35 (11)	C13—C14—H14	119.5
C4—C5—H5A	109.4	C14—C15—C16	120.15 (15)
C6—C5—H5A	109.4	C14—C15—H15	119.9
C4—C5—H5B	109.4	C16—C15—H15	119.9
C6—C5—H5B	109.4	C17—C16—C15	119.52 (14)
H5A—C5—H5B	108.0	C17—C16—H16	120.2
N1—C6—C7	110.02 (10)	C15—C16—H16	120.2
N1—C6—C5	108.40 (11)	C16—C17—C18	120.58 (14)
C7—C6—C5	112.75 (10)	C16—C17—H17	119.7
N1—C6—H6	108.5	C18—C17—H17	119.7
C7—C6—H6	108.5	C17—C18—C13	120.54 (14)
C5—C6—H6	108.5	C17—C18—H18	119.7
C8—C7—C12	117.66 (13)	C13—C18—H18	119.7
C8—C7—C6	121.30 (12)	C6—N1—C2	113.14 (10)
C12—C7—C6	121.04 (11)	C6—N1—H1	110.1 (9)
C7—C8—C9	120.97 (14)	C2—N1—H1	109.7 (9)
C7—C8—H8	119.5		
			
N1—C2—C3—C4	−56.90 (15)	C6—C7—C12—C11	179.08 (13)
C13—C2—C3—C4	−178.62 (11)	N1—C2—C13—C14	−50.66 (15)
C2—C3—C4—C5	53.73 (18)	C3—C2—C13—C14	70.16 (15)
C3—C4—C5—C6	−53.20 (18)	N1—C2—C13—C18	128.55 (12)
C4—C5—C6—N1	55.84 (15)	C3—C2—C13—C18	−110.63 (14)
C4—C5—C6—C7	177.89 (12)	C18—C13—C14—C15	−0.6 (2)
N1—C6—C7—C8	−130.46 (12)	C2—C13—C14—C15	178.63 (13)
C5—C6—C7—C8	108.40 (14)	C13—C14—C15—C16	0.3 (2)
N1—C6—C7—C12	50.58 (15)	C14—C15—C16—C17	0.3 (2)
C5—C6—C7—C12	−70.56 (16)	C15—C16—C17—C18	−0.6 (2)
C12—C7—C8—C9	−0.1 (2)	C16—C17—C18—C13	0.4 (2)
C6—C7—C8—C9	−179.06 (12)	C14—C13—C18—C17	0.25 (19)
C7—C8—C9—C10	0.1 (2)	C2—C13—C18—C17	−178.98 (12)
C8—C9—C10—C11	−0.2 (2)	C7—C6—N1—C2	174.32 (10)
C9—C10—C11—C12	0.2 (2)	C5—C6—N1—C2	−61.97 (13)
C10—C11—C12—C7	−0.2 (2)	C13—C2—N1—C6	−173.66 (10)
C8—C7—C12—C11	0.1 (2)	C3—C2—N1—C6	62.57 (14)

### Hydrogen-bond geometry (Å, º)

Cg1 and Cg2 are the centroids of the C13–C18 and C7–C12 rings, respectively.

**Table:**

*D*—H···*A*	*D*—H	H···*A*	*D*···*A*	*D*—H···*A*
C3—H3*A*···*Cg*1^i^	0.97	3.00	3.719 (2)	132
C10—H10···*Cg*1^ii^	0.93	3.01	3.760 (2)	139
C16—H16···*Cg*2^iii^	0.93	3.03	3.799 (2)	141

Symmetry codes: (i) −*x*+2, −*y*+1, −*z*+2; (ii) −*x*+1, −*y*+2, −*z*+1; (iii) *x*, *y*−1, *z*.
